# No Evidence of SARS-CoV-2 Circulation in Rome (Italy) during the Pre-Pandemic Period: Results of a Retrospective Surveillance

**DOI:** 10.3390/ijerph17228461

**Published:** 2020-11-16

**Authors:** Carlo Capalbo, Enrico Bertamino, Alessandro Zerbetto, Iolanda Santino, Andrea Petrucca, Rita Mancini, Rita Bonfini, Valeria Alfonsi, Stefano Ferracuti, Paolo Marchetti, Maurizio Simmaco, Giovanni Battista Orsi, Christian Napoli

**Affiliations:** 1 Sant’Andrea Hospital, University La Sapienza, via di Grottarossa 1035-1039, 00189 Rome, Italy; enrico.bertamino@uniroma1.it (E.B.); alessandro.zerbetto@uniroma1.it (A.Z.); iolanda.santino@uniroma1.it (I.S.); andrea.petrucca@ospedalesantandrea.it (A.P.); rita.mancini@uniroma1.it (R.M.); rbonfini@ospedalesantandrea.it (R.B.); valfonsi@ospedalesantandrea.it (V.A.); stefano.ferracuti@uniroma1.it (S.F.); paolo.marchetti@uniroma1.it (P.M.); maurizio.simmaco@uniroma1.it (M.S.); giovanni.orsi@uniroma1.it (G.B.O.); christian.napoli@uniroma1.it (C.N.); 2 Department of Molecular Medicine, University La Sapienza, viale Regina Elena 291, 00161 Rome, Italy; 3 Department of Public Health and Infectious Diseases, University La Sapienza, Piazzale Aldo Moro 5, 00185 Rome, Italy; 4 Department of Clinical and Molecular Medicine, University La Sapienza, via di Grottarossa 1035-1039, 00189 Rome, Italy; 5 Department of Human Neurosciences, University La Sapienza, Piazzale Aldo Moro 5, 00185 Rome, Italy; 6 Department of Neurosciences, Mental Health, and Sensory Organs, “Sapienza” University of Rome, via di Grottarossa 1035-1039, 00189 Rome, Italy; 7 Department of Medical Surgical Sciences and Translational Medicine, University La Sapienza, via di Grottarossa 1035/1039, 00189 Rome, Italy

**Keywords:** SARS-CoV-2, coronavirus, COVID-19, epidemiological and etiological surveillance, influenza-like syndrome, pneumonia

## Abstract

In March 2020, the World Health Organization (WHO) declared that the COVID-19 outbreak recorded over the previous months could be characterized as a pandemic. The first known Italian SARS-CoV-2 positive case was reported on 21 February. In some countries, cases of suspected “COVID-19-like pneumonia” had been reported earlier than those officially accepted by health authorities. This has led many investigators to check preserved biological or environmental samples to see whether the virus was detectable on dates prior to those officially stated. With regard to Italy, the results of a microbiological screening in sewage samples collected between the end of February and the beginning of April 2020 from wastewaters in Milan (Northern Italy) and Rome (Central Italy) showed presence of SARS-CoV-2. In the present study, we evaluated, by means of a standardized diagnostic method, the SARS-CoV-2 infection prevalence amongst patients affected by severe acute respiratory syndrome (SARI) in an academic hospital located in Central Italy during the period of 1 November 2019–1 March 2020. Overall, the number of emergency room (ER) visits during the investigated period was 13,843. Of these, 1208 had an influenza-like syndrome, but only 166 matched the definition of SARI as stated in the study protocol. A total of 52 SARI cases were laboratory confirmed as influenza: 26 as a type B virus, 25 as a type A, and 1 as both viruses. Although about 17% of the total sample had laboratory or radiological data compatible with COVID-19, all the nasopharyngeal swabs stored underwent SARS-CoV-2 RT-PCR and tested negative. Based on our result, it is confirmed that the COVID-19 pandemic spread did not start prior to the “official” onset in central Italy. Routine monitoring of SARI causative agents at the local level is critical for reporting epidemiologic and etiologic trends that may differ from one country to another and also among different influenza seasons. This has a practical impact on prevention and control strategies.

## 1. Introduction

On 31 December 2019, Chinese health authorities reported a cluster of pneumonia cases of unknown etiology in the city of Wuhan (Hubei province, China) and on 11 February, the World Health Organization (WHO) announced that the respiratory disease caused by 2019-nCoV had been officially named COVID-19 (coronavirus disease 2019) [1,2]. On 11 March 2020, after assessing the levels of spread and severity of the SARS-CoV-2 infection, the World Health Organization (WHO) declared that the COVID-19 outbreak recorded over the past months could be characterized as a pandemic. However, it is possible that, in many countries, the first cases of COVID-19 had their onset earlier than those officially reported by health authorities. This has led many researchers to check in preserved biological or environmental samples whether the virus was detectable on dates prior to those officially accepted, confirming the importance of screening for infectious diseases for both individual and public health benefits [3]. Deslandes et al. demonstrated, with collected respiratory samples, that SARS-CoV-2 was already spreading in France in late December 2019, at least 4 weeks before the first official cases in this country. Moreover, preliminary studies have reported the detection of SARS-CoV-2 RNA in wastewater in France, the Netherlands, the USA, and Australia and supported an earlier SARS-CoV-2 circulation than those officially accepted by health authorities [4]. With regard to Italy, the first known case was reported in February [1,2]. La Rosa et al. reported results of the screening for SARS-CoV-2 presence in sewage samples collected between the end of February and the beginning of April 2020 from wastewaters in Milan (Northern Italy) and Rome (Central Italy). One positive case was obtained from a Milan wastewater sample collected a few days after the first notified Italian case of autochthonous SARS-CoV-2 [5]. In regard to the same samples collected in Rome, viral RNA was detected on 31 March, when the epidemic had spread considerably in Italy. On that date, a total of 77,635 SARS-CoV-2 infections had been reported in Italy, of which 3095 occurred in the Lazio region and 2186 in the province of Rome [5]. Very recently, the same research team demonstrated that in the wastewaters of Northern Italy, there were already traces of the SARS-CoV-2 virus in December 2019. This was discovered by a study published by the Istituto Superiore di Sanità (ISS) carried out through the analysis of waste water collected in times prior to the onset of COVID-19 in Italy; no evidence of virus circulation was reported in Rome in that period [6,7].

From a diagnostic point of view, to date, as stated by both the Italian Ministry of Health and scientific data, the RNA RT-PCR assay of mucus obtained by nasopharyngeal swabs is considered to be the reference standard for the diagnosis of SARS-CoV-2 infection [8,9]. In the present study, we evaluated the SARS-CoV-2 infection prevalence using a standardized diagnostic method among patients affected by severe acute respiratory syndrome (SARI) in an academic hospital located in Central Italy, during the period of November 2019–March 2020, before the beginning of the epidemic in Italy. The study is aimed at obtaining evidence of the human circulation of SARS-CoV-2 before the onset of the first officially reported case in this region.

## 2. Materials and Methods

### 2.1. Study Population and Design

A cross-sectional study was performed at a teaching hospital in Rome (Central Italy), a secondary referral center with approximately 450 beds and 1,300,000 services provided per year (including those for both inpatients and outpatients). The study was partially based on the retrospective data (1 November 2019–1 March 2020) of a modified influenza surveillance project of nasopharyngeal swabs and an influenza vaccines effectiveness evaluation. The study consisted of a systematic daily screening of all admissions in patients with a severe acute respiratory syndrome (SARI). To this purpose, we defined a SARI case as a hospitalized patient with at least one systemic sign or symptom (fever or low-grade fever, headache, myalgia, or generalized malaise) or deterioration of general conditions (fatigue, weight loss, anorexia, or confusion and dizziness), and at least one respiratory sign or symptom (cough, sore throat, breathing difficulties) present at the time of admission or within 48 h after admission to the hospital [10].

To describe the characteristics of patients and explore risk factors, data were collected using a standardized questionnaire. From clinical records, the anamnestic data were also collected, if necessary, involving general practitioners.

The COVID-19 Reporting and Data System (CO-RADS) and COVID-19 lab-score were used for a standardized assessment of the pulmonary involvement of COVID-19 in non-enhanced chest CT and laboratory evaluations, respectively [11,12]. In particular, CO-RADS scores describe the categories and the corresponding level of suspicion for pulmonary involvement in COVID-19: CO-RADS 0 is chosen if none of the five categories can be assigned (insufficient image quality). CO-RADS 1 implies a very low level of suspicion for pulmonary involvement by COVID-19 based on either a normal CT or CT findings of unequivocal non-infectious etiology. CO-RADS 2 implies a low level of suspicion for pulmonary involvement by COVID-19. CO-RADS 3 implies equivocal findings for pulmonary involvement of COVID-19 based on CT features that can also be found in other viral pneumonias or non-infectious etiologies. CO-RADS 4 implies a high level of suspicion for pulmonary involvement by COVID-19 based on CT findings that are typical for COVID-19 but show some overlap with other pneumonias. Finally, CO-RADS 5 implies a very high level of suspicion for pulmonary involvement by COVID-19 based on typical CT findings (mandatory features are ground-glass opacities) [11].

Two nose–pharyngeal swabs were performed for each patient. The first one was immediately analyzed for influenza virus detection and the second one was stored for further analysis that might eventually be needed. All the second swabs were later tested for SARS-CoV-2 RT-PCR in the month of June 2020.

### 2.2. Laboratory Tests of SARS-CoV-2 Infection

SARS-CoV-2 RT-PCR was performed on mucus obtained from nasopharyngeal swabs using a commercial kit, according to the manufacturer’s instructions (Allplex™ 2019-nCoV Assay, Seegene Inc., Seoul, Korea). Molecular detection of SARS-CoV-2 RNA was performed following the manufacturer’s instructions. Briefly, 10 µL of internal control was added to each aliquot of 200 µL nasopharyngeal and oropharyngeal specimen subjected to viral RNA extraction, as previously mentioned. Subsequently, 8 out of 60 µL eluted RNA samples was RT-PCR amplified as suggested by the manufacturer on a Biorad CFX96 real-time system. The Allplex™ SARS-CoV-2 Assay is based on the simultaneous detection of the E gene (sabercovirus, β-coronavirus), RdRP and N genes (specific for SARS-CoV-2), and internal control. The interpretation criteria were as follows: a positive signal detected in both internal control and at least two of the three investigates genes (E, RdRP, and N) with a cycle threshold (CT) value ≤40 were considered positive for SARS-CoV-2; whereas, positive signals in internal control and only one gene with a CT value ≤40 were considered indeterminate and subjected to a second nasopharyngeal and oropharyngeal sampling. All other results were considered negative. The declared load of detection (LoD) was 100 RNA copies/reaction. Moreover, we tested whether the detection system worked well when the assay was firstly implemented in the routine of the hospital laboratory. To this purpose, a serial ten-fold dilution of a positive sample, which presented an amplification of the N and E SARS-CoV-2 genes, was performed. The positive sample and all dilutions were analyzed by the detection assay in triplicate. Results confirmed that the assay was able to detect SARS-CoV-2 RNA within the limits of detection, and these results were reproducible. 

Type A and B influenza viruses were detected using rapid chromatographic tests (Rapid Influenza A + B test, Immunospark, Pomezia RM, Italy).

### 2.3. Statistical Analyses

Descriptive statistics were expressed by number (percentage) of total, mean ± SD, and median (range).

### 2.4. Ethical Issue

The study was performed following the World Medical Association Declaration of Helsinki and did not include any identifiable human data. Approval from the Sapienza University of Rome Ethical Committee was obtained twice: for the first study of the seasonal SARI surveillance (n. 5544_2019 in November 2019), and for the second one related to the screening protocol for SARS-CoV-2 (n. 5773_2020 in April 2020).

## 3. Results 

Overall, the number of emergency room (ER) visits during the investigated period was 13,843. Of these, 1208 had symptoms consistent with an influenza-like syndrome, but only 166 matched the definition of SARI stated by the study design. A total of 52 (31.3%) SARI cases were confirmed as influenza: 26 as a type B virus, 25 as a type A, and 1 as both viruses. Out of 166 SARI cases, about 78% and 86% presented fever or malaise, respectively. Of them, 25% had been vaccinated in previous seasons. The mean age of SARI patients was 37.4 years. Most of them were male (54.8%), and about 7% were older than 85 years. Heart and lung diseases were the main associated comorbidities. The general characteristics of the enrolled patients (demographic, vaccination status, and clinical conditions) are reported in Table 1. No samples tested positive for SARS-CoV-2. In particular, the negative subgroup for influenza with highly suspect clinical, radiological (CO-RADS >4), and hematological parameters for COVID-19 was also negative (about 4%), confirming that these investigational parameters alone or combined are not sufficient to diagnose a SARS-CoV-2 infection.

## 4. Discussion

SARI are currently a major public health burden and in a great part they are caused by Influenza viruses, whose cases account for up to 50 million disease episodes and 72,000 deaths in Europe each year [13]. The consequences of SARI infection can be severe, both for the individual and for the healthcare system. The severity of the infection depends on the virus type/subtype, on host characteristics (e.g., age), and on other factors, such as access to care. Complications of SARI, such as pneumonia, are more common among specific risk groups, including elderly individuals, children under one year of age, and people affected with immune deficiencies [13]. Therefore, routine monitoring of infectious etiologies associated with SARI at the local level is critical for signaling epidemiologic and etiologic trends that may differ from one country to another, and also within the same country. During the last SARI season (2019–2020), apart from influenza viruses, a novel coronavirus belonging to the family Coronaviridae was responsible for the outbreak of a series of acute atypical severe respiratory tract syndrome whose first cases were reported in Wuhan, China [1,2]. It is believed that SARS-CoV-2 has zoonotic origins and a close genetic similarity to bat coronaviruses, suggesting it emerged from a bat-borne virus. The wet markets in the principal city of Wuhan are assumed to have been the specific causative locus of the sudden explosion of infections, and about three months after the COVID-19 pneumonia outbreak in the Chinese province of Hubei, Italy was affected by the SARS-CoV-2 pandemic [14]. However, several recent findings that are now coming to light show that this interpretation of the origin of the pandemic is overly simplified [5]. The epidemiologic differences noted across the affected countries surely highlight the different distribution of several factors such as demographics, climate, comorbidities, and many other features, making the interpretation and comparison of data difficult. A number of variants of the coronavirus would in principle have had the ability to initiate the pandemic well before January of the current year [15]. In Europe, cases of suspected pneumonia had been reported earlier than December 2019, and Deslandes et al. reported a case of a patient hospitalized (Northern Paris) in December 2019 in an intensive care unit for hemoptysis and an influenza-like illness, with no etiological diagnosis [4]. In this study, researchers took 14 samples from the hospital’s bank of respiratory samples taken from hospitalized patients. The chosen samples were from patients admitted to the hospital’s intensive care unit between 2 December 2019 and 16 January 2020, and who had an influenza-like illness and ground glass opacity. These samples underwent RT-PCR testing for SARS-CoV-2, and one came back positive. Based on this result, it appears plausible that the COVID-19 epidemic started much earlier in France [4]. Similarly to other countries, in Italy, La Rosa et al. have identified the presence of SARS-CoV-2 in wastewater collected in times prior to the onset of COVID-19 in Italy. One of the positive cases was obtained in the Northern region (Lombardy) wastewater sample, collected a few days after the first notified Italian autochthonous SARS-CoV-2 case. As regards the samples collected in a low incidence area (Lazio), SARS-CoV-2 was detected on 31 March, when the epidemic had spread considerably in Italy [5,6,7]. To the best of our knowledge, no study similar to that of Deslandes et al. has been conducted in Italy to date. In Lazio, the regional hospital network for COVID-19 management was early divided into hub-and-spoke structures, and the hospital involved in the present study was one out of five COVID-19 hubs in Rome. During the pandemic spread, about 70% of all SARS-CoV-2 positive patients in Lazio were treated in Rome (a total of 2186 SARS-CoV-2 infections had been reported in the province of Rome) [5,16]. In the present study, we retrospectively evaluated the SARS-CoV-2 infection prevalence among patients affected by SARI by means of a standardized diagnostic method in an academic hospital located in Central Italy. Although the sample had laboratory or radiological data compatible with COVID-19, all the nasopharyngeal swabs stored underwent SARS-CoV-2 RT-PCR and tested negative. 

In this study, A and B type influenza viruses were isolated among patients with SARI with the same prevalence (about 50% each). This is not in line with previously reported findings of the previous influenza season (2018/19) in people with laboratory-confirmed influenza, where Type B influenza was uncommon [13]. Circulating influenza viruses vary from season to season and influenza vaccines are reformulated regularly on this base. This means that the surveillance of etiological nature or influenza cases has a great impact on annual influenza vaccine composition. Seasonal vaccination is considered the most effective way to prevent influenza and its complications, with a recommended target of a 75% coverage among groups at high risk of infection. In Italy, annual vaccination is provided free of charge for individuals who are at high risk, including those aged ≥65 years, healthcare workers, people affected by chronic diseases and immune deficiencies, and pregnant women [13]. In 2018/19, around 15.8% of the Italian population overall and 53.1% of people ≥65 years received a seasonal influenza vaccine [13]. This year, adherence to influenza vaccination is fundamental to also reduce SARIs other than COVID-19. The Lazio region has, for this reason, imposed compulsory vaccination for influenza for all healthcare workers. 

The present study has some main limitations. First of all, this was a small single-center study conducted in Rome, probably not able to reflect the spread of infection in Central Italy. Moreover, due to the retrospective design of the study, the sample is not well balanced in terms of gender and the nasopharyngeal swabs for the SARS-CoV-2 infection were performed once over a period of four months. Therefore, we were not able to exclude the possibility that patients who tested negative at the beginning of the testing period might have become positive over time. Lastly, although as stated by the Italian Ministry of Health, the RNA RT-PCR assay of mucus obtained by nasopharyngeal swabs is considered the reference standard for the diagnosis of SARS-CoV-2 infection [9], the possibility of false-negative results due to the sensitivity of RT-PCR in collected samples may have affected the results of the study. 

However, our study does not support the thesis relating to the onset of co-suspected pneumonia before the official onset date.

## 5. Conclusions

In conclusion, despite the limits of the study, such as the sample size, the retrospective nature of the analysis, and the sensitivity of RT-PCR, it appears plausible that the COVID-19 pandemic spread did not start much earlier, prior to the “official” onset in central Italy. Routine monitoring of SARI causative agents at the local level is critical for the report of epidemiologic and etiologic trends that may differ from one country to another, and also among different influenza seasons. This has a practical impact on prevention and control strategies.

## Figures and Tables

**Table 1:** Characteristics of the enrolled patients.

Characteristics	November 2019–March 2020 SARI Cases (N = 166)
	N (%)
Age (Mean)	37.4
Aged 85+ years	12 (7.2)
Sex = male	91 (54.8)
N° hospitalization in past 12 months (Mean ± SE)	0.61 ± 0.10
Influenza (type A)	25 (15%)
Influenza (type B)	26 (15.6%)
Influenza (type A and B)	1 (0.6%)
**Vaccination Status**	
Season 2019–2020	43 (25.9)
Season 2018–2019	41 (24.7)
Season 2017–2018	42 (25.3)
**Underlying Conditions**	
Diabetes	23 (15.7)
Heart disease	57 (34.3)
Lung disease	56 (33.7)
Liver disease	1 (0.6)
Immune suppressed	3 (1.8)
Cancer	20 (12)
Renal disease	9 (5.4)
Dementia or stroke	4 (2.4)
Rheumatologic disease	0 (0.0)
Obese	11 (6.6)
**Symptoms**	
Feverishness or fever	129 (77.7)
Malaise	143 (86.1)
Headache	52 (31.3)
Myalgia	59 (35.5)
Cough	143 (86.1)
Sore throat	81 (48.8)
Short breath	122 (73.5)
Loss of smell and/or taste	0
**Scores**	
Covid19 Lab-score Log Percent >50%	28 (17)
Covid19 Lab-score Log Percent >50% plus CO RAD >4	7 (4.2)

## References

[1] Wang C., Horby P.W., Hayden F.G., Gao G.F. (2020). A novel coronavirus outbreak of global health concern. Lancet.

[2] Tian Y., Rong L., Nian W., He Y. (2020). Review article: Gastrointestinal features in COVID-19 and the possibility of faecal transmission. Aliment. Pharmacol. Ther..

[3] Napoli C., Dente M.G., Kärki T., Riccardo F., Rossi P., Declich S., Fccbhtmbbs N. (2015). Screening for Infectious Diseases among Newly Arrived Migrants: Experiences and Practices in Non-EU Countries of the Mediterranean Basin and Black Sea. Int. J. Environ. Res. Public Health.

[4] Deslandes A., Berti V., Tandjaoui-Lambotte Y., Alloui C., Carbonnelle E., Zahar J., Brichler S., Cohen Y. (2020). SARS-CoV-2 was already spreading in France in late December 2019. Int. J. Antimicrob. Agents.

[5] La Rosa G., Iaconelli M., Mancini P., Ferraro G.B., Veneri C., Bonadonna L., Lucentini L., Suffredini E. (2020). First detection of SARS-CoV-2 in untreated wastewaters in Italy. Sci. Total Environ..

[6] Istituto Superiore di Sanità CS N°39/2020—Studio ISS su Acque di Scarico, a Milano e Torino Sars-Cov-2 Presente già a Dicembre. https://www.iss.it/comunicati-stampa1.

[7] La Rosa G., Mancini P., Ferraro G.B., Veneri C., Iaconelli M., Bonadonna L., Lucentini L., Suffredini E. (2020). SARS-CoV-2 has been circulating in northern Italy since December 2019: Evidence from environmental monitoring. Sci. Total Environ..

[8] Italian Ministry of Health (2020). Covid-19, Pandemia di COVID-19—Aggiornamento delle Indicazioni sui Test Diagnostici e sui Criteri da Adottare Nella Determinazione delle Priorità. Aggiornamento delle Indicazioni Relative alla Diagnosi di Laboratorio. http://www.trovanorme.salute.gov.it/norme/renderNormsanPdf?anno=2020&codLeg=73799&parte=1%20&serie=null.

[9] Lahner E., Dilaghi E., Prestigiacomo C., Alessio G., Marcellini L., Simmaco M., Santino I., Orsi G.B., Anibaldi P., Marcolongo A. (2020). Prevalence of Sars-Cov-2 Infection in Health Workers (HWs) and Diagnostic Test Performance: The Experience of a Teaching Hospital in Central Italy. Int. J. Environ. Res. Public Health.

[10] Bella A., Gesualdo F., Orsi A., Arcuri C., Chironna M., Loconsole D., Napoli C., Orsi G.B., Manini I., Montomoli E. (2019). Effectiveness of the trivalent MF59 adjuvated influenza vaccine in preventing hospitalization due to influenza B and A(H1N1)pdm09 viruses in the elderly in Italy, 2017–2018 season. Expert Rev. Vaccines.

[11] Prokop M., Van Everdingen W.M., Vellinga T.V.R., Van Ufford H.Q., Stöger L., Beenen L.F.M., Geurts B., Gietema H., Krdzalic J., Schaefer-Prokop C. (2020). CO-RADS: A Categorical CT Assessment Scheme for Patients Suspected of Having COVID-19—Definition and Evaluation. Radiology.

[12] Covid 19 Labscore. https://genom-analyse.de/labsave.

[13] Rizzo C., Gesualdo F., Loconsole D., Pandolfi E., Bella A., Orsi A., Guarona G., Panatto D., Icardi G., Napoli C. (2020). Moderate Vaccine Effectiveness against Severe Acute Respiratory Infection Caused by A(H1N1)pdm09 Influenza Virus and No Effectiveness against A(H3N2) Influenza Virus in the 2018/2019 Season in Italy. Vaccines.

[14] Gagliano A., Villani P.G., Co’ F.M., Manelli A., Paglia S., Bisagni P.A.G., Perotti G., Storti E., Lombardo M. (2020). COVID-19 Epidemic in the Middle Province of Northern Italy: Impact, Logistics, and Strategy in the First Line Hospital. Disaster Med. Public Health Prep..

[15] Platto S., Xue T., Carafoli E. (2020). COVID19: An announced pandemic. Cell Death Dis..

[16] Capalbo C., Aceti A., Simmaco M., Bonfini R., Rocco M., Ricci A., Napoli C., Rocco M., Alfonsi V., Teggi A. (2020). The Exponential Phase of the Covid-19 Pandemic in Central Italy: An Integrated Care Pathway. Int. J. Environ. Res. Public Health.

